# Mutations in the *CYP27B1* gene cause vitamin D dependent rickets in pugs

**DOI:** 10.1111/jvim.16791

**Published:** 2023-06-09

**Authors:** Cecilia Rohdin, Chao Wang, Gustaf Brander, Veronica Rondahl, Åsa Karlsson, Lisa Friling, Anthony Fischetti, Jennifer Meadows, Jens Häggström, Karin Hultin Jäderlund, Ingrid Ljungvall, Kerstin Lindblad‐Toh

**Affiliations:** ^1^ Department of Clinical Sciences Swedish University of Agricultural Science Uppsala Sweden; ^2^ Anicura Albano Small Animal Hospital Danderyd Sweden; ^3^ Department of Medical Biochemistry and Microbiology Science for Life Laboratory Uppsala Sweden; ^4^ Broad Institute of MIT and Harvard Cambridge Massachusetts USA; ^5^ Biovet Sollentuna Sweden; ^6^ AniCura Djursjukhuset Albano Ringgold Standard Institution Danderyd Sweden; ^7^ Department of Diagnostic Imaging, Animal Medical Center New York New York USA; ^8^ Department of Companion Animal Clinical Sciences Norges Miljo‐ og Biovitenskapelige Universitet Fakultet for Veterinarmedisin og Biovitenskap Oslo Norway

**Keywords:** bone, hypocalcemia, pug, vitamin D

## Abstract

Rickets is a disorder of bone development and can be the result of either dietary or genetic causes. Here, related pugs from 2 litters were included. Three pugs had clinical signs including, lameness, bone deformities, and dyspnea. One other pug was found dead. Radiographs of 2 affected pugs, 5 and 6 months old, showed generalized widening, and irregular margination of the physes of both the appendicular and the axial skeleton with generalized decrease in bone opacity and bulbous swelling of the costochondral junctions. Two pugs had low serum calcium and 1,25 (OH)_2_D_3_ concentrations. Test results further indicated secondary hyperparathyroidism with adequate concentrations of 25‐hydroxyvitamin D. Necropsy revealed tongue‐like projections of cartilage extending into the metaphysis consistent with rickets, loss of metaphyseal mineralization and lung pathology. Vitamin D‐dependent rickets was diagnosed. A truncating mutation in the 1α‐hydroxylase gene (*CYP27B1*) was identified by genome sequence analysis of the pugs with VDDR type 1A. Vitamin D‐dependent rickets type 1A can occur in young pugs, and if left untreated is a life‐threatening condition. Early medical intervention can reverse clinical signs and should be instituted as soon as possible.

**Abbreviations**
ALP: alkaline phosphatase
CT: computed tomography
PTH: parathyroid hormone
PU/PD: polyuria and polydipsia
VDDR: vitamin D‐dependent rickets
VDR: vitamin D receptor
WGS: whole genome sequencing

## 1 INTRODUCTION

Absence of calcitriol in young, growing animals results in rickets. In dogs, rickets is diagnosed as a consequence of acquired (dietary) or congenital (genetic) vitamin D disorders.^1^, ^2^, ^3^, ^4^, ^5^, ^6^, ^7^ Genetic disorders of vitamin D occur from a young age in dogs with a history of adequate vitamin D intake and can be classified into 3 types. Vitamin D‐dependent rickets (VDDR) type 1A is caused by an impaired conversion of 25 (OH)D_3_ to 1,25‐dihydroxyvitamin D_3_. Vitamin D‐dependent rickets type 1B is caused by a failure of conversion from vitamin D to 25 (OH)D_3_. The third type, type 2A, involves the vitamin D receptor (VDR) gene and is called hereditary resistant rickets.^6^, ^8^

Vitamin D‐dependent rickets in dogs are rarely described in the literature and include 1 female 3‐month‐old St Bernard with suspected VDDR type 1A and a female 5‐month‐old Pomeranian with confirmed VDDR type 2A.^9^, ^10^ In people, genetic disorders of vitamin D are rare autosomal recessive disorders that present with rickets in infancy. Mutations in the *CYP27B* gene are recognized as the molecular basis for the 1α‐hydroxylase deficiency causing VDDR type 1A.^11^

This case report describes VDDR type 1A in related pugs.

## 2 CASE DESCRIPTIONS

**FIGURE 1:**
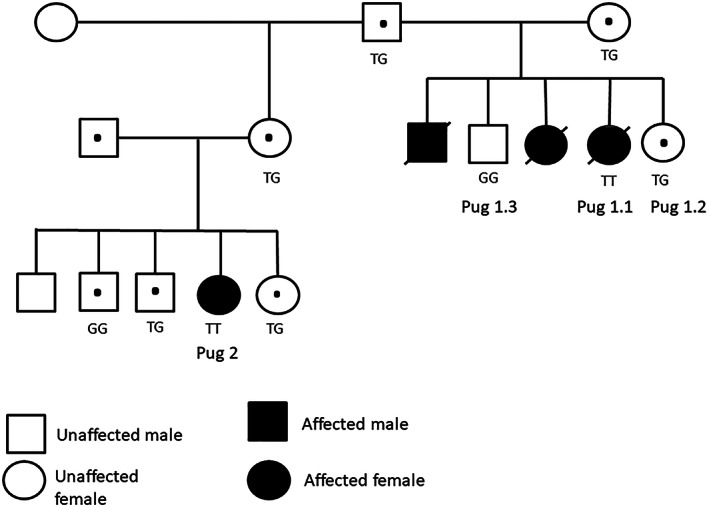
Family pedigree of litter 1 and litter 2. Filled circles (female) or squares (male) indicate homozygous (TT) or dead subjects; circles and squares with dots indicate heterozygous subjects (GT); and empty circles and squares indicate unaffected (GG) (n = 2) or not genetically investigated (n = 3) subjects.

Four young pugs from 2 closely related litters (litter 1 and 2, comprising a total of 10 pugs) were included (Figure 1). Affected individual pugs will be referred to as Pug 1.1^♀^, Pug 1.2^♀^, Pug 1.3^♂^ and Pug 2^♀^. Pug 1.1^♀^, Pug 1.2^♀^, and Pug 1.3^♂^, were siblings. The owner of Pug 1.1^♀^ was also the breeder of litter 1. All puppies were on a nutritionally balanced commercial dog food for growing dogs. Two female pugs, Pug 1.1^♀^ and Pug 2^♀^ presented at different occasions, with progressive signs of a valgus deformity of the thoracic limbs, abnormal spinal curvature, and reluctance to walk. Both were examined by the first author. Pug 1.1^♀^ presented at 6 months of age. The dog had, according to the owner, developed normally up to 13 weeks of age, before bone deformities were first recognized. Upon presentation Pug 1.1^♀^ was severely lethargic, reluctant to move, bloated, and moderately‐severely dyspneic. Thoracic and pelvic limbs were externally rotated from the carpal and tarsal joint respectively. In addition, thoracic kyphosis was present. The dog had muscle weakness but her neurological stature could not reliably be assessed because her lethargy and unwillingness to move. The owner elected euthanasia for the dog, and decided to donate the body for necropsy and further investigation of the underlying etiology. According to the breeder of litter 1, 2 siblings, 1.2^♀^ and 1.3^♂^, were already dead, both had been sent for necropsy without any diagnostic workout performed. Pug 1.2^♀^, the female sibling, died unexpectedly during sleep at 4 months of age. The dog had not shown any clinical abnormalities before her death. Pug 1.3^♂^, the male sibling, presented with acute dyspnea at 5.5 months of age. According to the owner the dog had shown signs of pelvic limb lameness before the onset of respiratory signs. Because of the severity of the dyspnea the puppy was euthanized without any diagnostic workup. Pug 2^♀^ presented at 5 months of age because of a shorter stature compared with her littermates. The dog was alert but less active with a mild valgus deformity of the thoracic limbs, and presented with a bilateral thoracic limb lameness and painful physes. None of her 4 littermates showed any clinical signs or bone deformities.

## 3 DIAGNOSTIC FINDINGS

Pug 1.1^♀^ and 2^♀^ both showed hypocalcemia (1.6 and 1.6 mmol/L, respectively; healthy age‐matched sibling 2.8 mmol/L) and markedly low concentrations of serum 1,25‐dihydroxyvitamin D_3_ (analyzed at the Veterinary Diagnostic Laboratory, Michigan State University, USA, using a radioimmunoassay [RIA]; 9 and 8 pmol/L respectively; healthy age‐matched sibling 566 pmol/L). Whereas abnormalities of 25 (OH)D_3_ (analyzed at IDEXX Laboratories, Germany, using a high‐performance liquid chromatography [HPLC]) concentrations (80 and 232 nmol/L respectively; healthy age‐matched sibling 57 nmol/L) were not detected, the parathyroid hormone concentration (analyzed at the Nationwide Specialist Laboratories, London, UK, using a radioimmunoassay [RIA]) of Pug 2 (113 pg/mL) and the alkaline phosphatase (ALP) activities (7.6 μkat/L and 6.1 μkat/L respectively; healthy age matched sibling 1.7 μkat/L) were high. The separated EDTA plasma was transported frozen.

**FIGURE 2:**
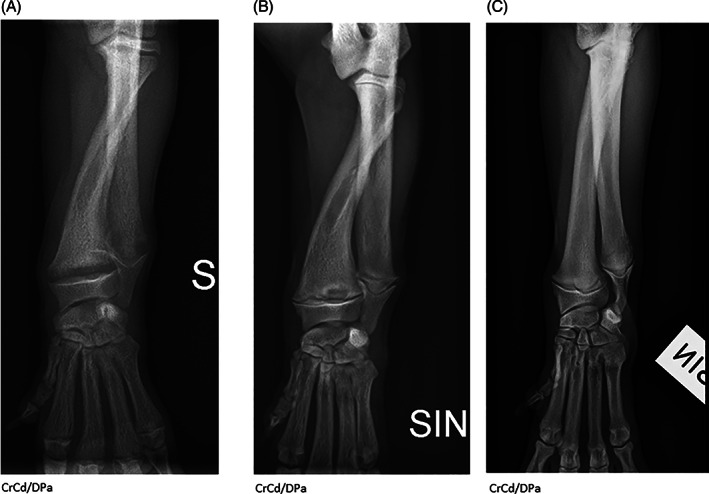
Radiographic examinations of the antebrachium and carpus (CrCd/DPa—projections) before and after initiation of corrective treatment with 1,25‐dihydroxyvitamin D3, (Etalpha). The physeal widening, irregular marginations of the metaphyses, and osteopenia improve over time. Radiographic changes are most dramatic in the distal radius and ulna. There is unbalanced growth between the radius and ulna resulting in elbow joint incongruity with secondary subchondral sclerosis. (A) Pug 2 with VDDR type 1A before treatment. (B) Pug 2 with VDDR type 1A after 6 weeks on treatment with 1,25‐dihydroxyvitamin D3 (Etalpha). (C) Age‐matched control pug (the dog has an unrelated defect in the elbow joint).

**FIGURE 3:**
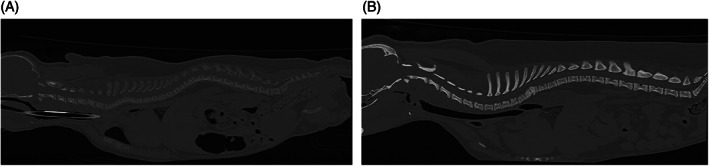
(A) Computed tomography images of the entire spine of Pug 1.1♀ with VDDR type 1A showing generalized decrease in bone density (attenuation). Endplate widening is also noted. (B) Computed tomography images of the entire spine of an age‐matched control pug.

Radiographs of the thoracic limbs of Pug 1.1^♀^ and 2^♀^ (Figure 2) revealed generalized widening of the physes, most notably the distal radial and ulnar physes bilaterally. The proximal margin of the widened physis had an irregular margination. In the pelvic limbs, similar widening of the physes was noted in the proximal/distal femur and proximal tibia bilaterally. Irregular physeal margin adjacent to the metaphysis was present though not as prominent as in the distal radius/ulna. Similar radiographic findings were seen in physes of both the appendicular and the axial skeleton, with generalized decrease in bone opacity and bulbous swelling of the costochondral junctions. The radiographic findings were most consistent with a metabolic bone disease, more specifically, hypovitaminosis D (“rickets”). In both pugs computed tomography (CT) of the entire spine revealed endplate physes that were generally widened and the vertebral column was undulating with kyphosis and marked loss of bone density (attenuation; Figure 3). The lungs of Pug 1.1 showed severe bilateral increase in opacity. The presence of an alveolar pattern, more pronounced ventrally, suggested pneumonia.

## 4 NECROPSY FINDINGS

**FIGURE 4:**
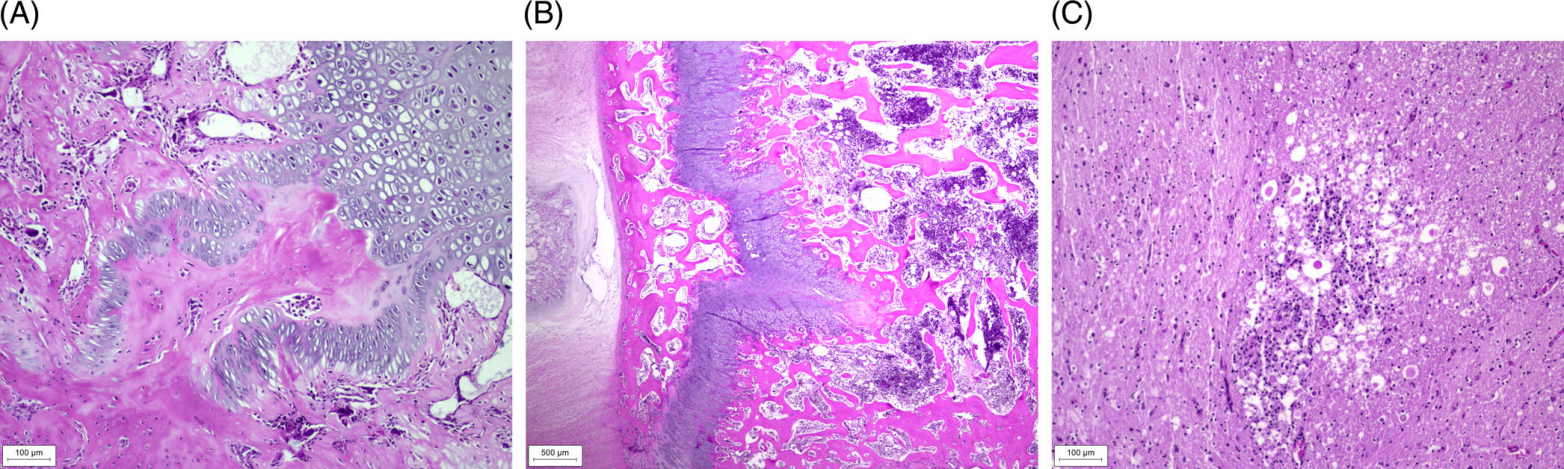
(A) Histopathological changes at sites of enchondral ossification in the bones of Pug 1.1♀ with VDDR type 1A. Costochondral joint, longitudinal section stained with hematoxylin and eosin (H&E), showing retention of hypertrophic chondrocytes and tongue‐like projections of cartilage extending into the metaphysis from the physeal cartilage. (B) Histopathological changes at sites of enchondral ossification in the bones of Pug 1.1♀ with VDDR type 1A. Vertebrae T13–L1, transverse section (H&E), showing disorganized columns of hypertrophic chondrocytes and tongue‐like projections of cartilage in the metaphysis. (C) Spinal cord at T13–L1, transverse section (H&E), showing focal malacia with moderate parenchymal destruction and gliosis of primarily the ventral funiculi at the level of spinal cord stenosis.

Pug 1.1^♀^ and Pug 1.3^♂^ underwent necropsy using a protocol focusing specifically on skeletal histopathology. Pug 1.2 underwent standard necropsy. The costochondral junctions of Pug 1.1^♀^ and Pug 1.3^♂^ were enlarged with tongues of cartilage extending into the metaphysis and disruption of the normal architecture of the primary spongiosa (Figure 4). Similar but less pronounced changes, with tongue‐like projections of cartilage extending into the metaphyses, were found in the selected long bones.

Corresponding to the CT findings in Pug 1.1^♀^, the 13th thoracic and the first lumbar vertebrae showed prominent deformation with kyphosis and narrowing of the vertebral canal and stenosis of the spinal cord. In the adjacent spinal cord there was focal malacia with moderate destruction of the parenchyma mainly involving the ventral funiculi (Figure 4). In the lungs of pug 1.1^♀^, a fibrinous bronchopneumonia was confirmed. Pug 1.2^♀^, which unexpectedly died at 4 months of age, presented with extensive pulmonary hemorrhage without identifiable etiology and a mild to moderate interstitial pneumonia in the right cranial lobe. In Pug 1.3^♂^, severe autolysis made it impossible to reliably evaluate the lungs. The skeletal structures of Pug 1.2^♀^ were not examined, because at the time there was no clinical suspicion of metabolic bone disease.

## 5 CLINICAL MANAGEMENT OF PUG 2^♀^

When Pug 2^♀^ was confirmed with hypocalcemia and a low concentration of 1,25‐dihydroxyvitamin D_3_, accompanied by similar radiographic abnormalities as in Pug 1.1^♀^, corrective treatment with 1,25‐dihydroxyvitamin D_3_, (Etalpha) 0.5 μg/day per oral q24h^9^ was initiated. After 2 weeks of treatment the total serum calcium had normalized (2.5 mmol/L). Although the pug was still reluctant to move, radiographs showed improvement with less widened physes and increased mineralization. To reach the same serum calcium level in the blood as healthy pugs of similar age, the dose was increased to 0.7 μg/day of Etalpha per oral. Six weeks after the start of treatment with 1,25‐dihydroxyvitamin D_3_ the puppy was bright, active, and although still stunted in growth her bone axis was straight and she showed no pain on palpation of the physes (Figure 2). Corrective treatment with Etalpha was continued for a total of 2.5 months when polyuria and polydipsia (PU/PD) developed. Without a further change in the dose of treatment, or loss of weight, the serum calcium had increased to 3.3 mmol/L. Because of the PU/PD and the serum calcium concentration, the corrective treatment was discontinued and the serum calcium normalized within a week (2.6 mmol/L). The sudden increase in serum calcium without corresponding change in dose of administered 1,25‐dihydroxyvitamin D_3_ occurred as Pug 2^♀^ came into estrus. Etalpha was again instituted at a dose of 0.4 to 0.5 μg/week per oral. Upon follow up at 22 months of age the dog was normocalcemic with an adequate 1,25‐dihydroxyvitamin D_3_ concentration (145 pmol/L).

## 6 GENETIC INVESTIGATIONS

Whole genome sequencing (WGS) data was generated (SNP&SEQ Technology Platform in Uppsala, part of the National Genomics Infrastructure [NGI] Sweden and Science for Life Laboratory) from the 2 rickets pugs, Pug 1.1 and Pug 2, and the variants in the candidate gene, *CYP27B1* were screened using 20 pugs from previous studies^12^ as controls. Two variants that fit the pattern of homozygosity in cases, but not in control dogs, were identified from *CYP27B1*: chr10:2184310G>A in the 5′UTR, and chr10:2182971G>T in a coding exon. Notably, the chr10:2182971G>T was predicted to be “high impact” by SNPeff, and causes a stop mutation (c.261C>A; XM_038549826.1) at the 87 codon of *CYP27B1*. This premature truncation leads to the loss of 83% of the protein sequence (508 codons for normal *CYP27B1*), which contains a large part of the P450 conserved domain (41‐505 codons; NCBI CCD: pfam00067).

**FIGURE 5:**
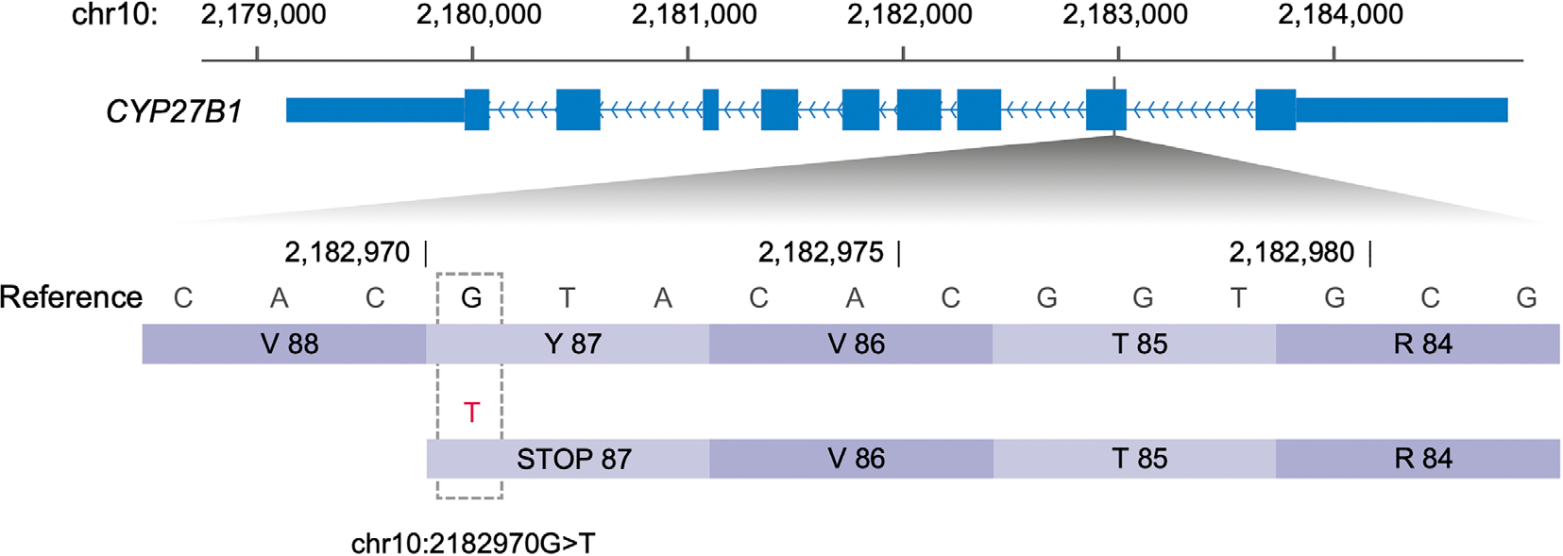
A stop gain mutation (chr10:2182971G>T) in CYP27B1 identified from the 2 pugs (Pug 1.1 and Pug 2) with VDDR type 1A. This premature truncation occurs at the 87th codon in exon 2, which leads to 83% of the protein sequence to be missing.

The mutation was genotyped in 8 additional pugs from the same pedigree with Sanger sequencing (Eurofins Genomics, Germany). The genotype of the 8 pugs from the same pedigree (Figure 1) perfectly matched the segregation of the rickets phenotype (Figure 5). The mutated allele T was not observed in the 20 control pugs, or other purebred dogs from a large population study.^13^

## 7 DISCUSSION

In this study we identified a putative causative mutation for VDDR type 1A in related pugs. In addition, our study illustrates the unique radiographic characteristics, and the skeletal histological features of rickets. Although in people there are many mutations of the *CYP27B1* gene^14^, ^15^, ^16^, ^17^, ^18^, ^19^, ^20^ VDDR type 1A is considered a rare disorder,^11^, ^21^ and has previously not been confirmed in dogs. In people, no variant was reported at the corresponding position. However, next to the position, a 1‐base deletion was reported in French Canadian people with VDDR type IA.^16^ Children with 1α‐hydroxylase deficiency, caused by mutations in the *CYP27B1* gene, present with a clinical picture of joint pain and deformity, hypotonia, muscle weakness, growth failure, and sometimes hypocalcemic seizures or fractures.^11^ Children with VDDR type 1A further present with similar laboratory abnormalities as the 2 rickets pugs presented here: hypocalcemia, secondary hyperparathyroidism, elevated ALP, and low or undetectable 1,25‐dihydroxyvitamin D_3_ in the presence of adequate 25(OH)D_3_ levels.^11^, ^21^, ^22^, ^23^, ^24^ Interestingly, whereas the affected pugs presented with marked bone affection, none presented with seizures.

Bone tissue develops either by intramembranous or enchondral ossification. Enchondral ossification is responsible for the formation of long bones of the appendicular skeleton (limbs) and the axial skeleton (vertebras and ribs). The enchondral bone formation is dependent on calcium, phosphate, and vitamin D for the terminal differentiation of chondrocytes to osteocytes.^25^ Rickets is a primarily skeletal disease that causes soft bones, deformed legs, and stunted growth. In rickets, cartilage in the physes continues to accumulate but cannot undergo normal enchondral ossification, leading to the characteristic radiographic findings of widening of the physes. The corresponding microscopic changes include persistent tongues and islands of hypertrophic chondrocytes surrounded by unmineralized osteoid of the physes, as was seen to a variable degree in Pug 1.1^♀^ and her male sibling, Pug 1.3^♂^. These changes can be seen at any physes in affected individuals but are always most dramatic in the bones where the growth is the greatest, for example, in the distal radius and ulna. Consequential changes such as unbalanced growth of paired bones and bowing of long bones can also occur.^26^

Although rickets was not confirmed in Pug 1.2^♀^, this dog presented with pneumonia and pulmonary hemorrhage. With rickets the lungs are the second, after the skeleton, common organ affected by vitamin D deficiency.^27^ Pulmonary hemorrhage, the presumed cause of death of Pug 1.2^♀^, has however only rarely been described in rickets.^28^ Pneumonia, also confirmed in Pug 1.1^♀^, is a common finding in children with vitamin D deficiency because of the important role of vitamin D in the function of the immune system.^27^, ^29^ Respiratory manifestations of rickets could develop from impaired clearance and infections either as a consequence of muscular hypotonia, involving the diaphragm and intercostal muscles, or from a soft thoracic rib cage causing compression of the lung parenchyma.^30^

Both Pug 1.1^♀^ and Pug 2^♀^ presented with a kyphotic malalignment of the thoracic spine. Kyphosis is usually the consequence of vertebral malformations which are commonly seen in dogs of this breed.^31^ Obvious vertebral malformations were not confirmed by CT examination in the 2 affected pugs. However, necropsy of Pug 1.1^♀^ showed severe deformation of the 13th thoracic and first lumbar vertebrae with defects in mineralization, a narrowed vertebral canal, and adjacent vertebrae showed similar mild‐moderate lesions. Associated with the thoracic kyphotic malalignment and the spinal cord stenosis there was focal spinal cord myelomalacia. We can only speculate whether these findings in the vertebrae and spinal cord were incidental, related to the hypovitaminosis D,^26^, ^32^ or potentially represented an early variant of the vertebral and spinal cord lesions commonly seen in older pugs.^33^

The need for corrective treatment with 1,25‐dihydroxyvitamin D_3_ ceased as Pug 2^♀^ matured and came in heat, estrus. Estrogen plays an important role in the growth and maturation of bone and is needed for closure of the growth plates.^34^ The closure of the physes and termination of the skeletal growth reduces the requirement of 1,25‐dihydroxyvitamin D_3_ to maintain bone homeostasis. The signs of disease were obvious in Pug 1.1^♀^ and Pug 2^♀^ but it is possible that a milder form of the disease, as described in people,^15^, ^16^, ^19^ with a less disturbed ossification, remains undiscovered during growth in dogs of this breed. These bony lesions could remain silent or could cause clinical signs later in life. In people with VDDR type 1A shows some phenotypic variation, including those of mild phenotype, and it has been suggested that some mutants retain partial enzymatic activity.^16^ The truncation mutation of VDDR type 1A pugs, occurring at the beginning of the *CYP27B1* gene, probably caused the complete loss of a functional 1α‐hydroxylase protein. This could make these pugs a perfect animal model of *CYP27B1* inactivation for the studies of the VDDR type 1A disorders in people.

## CONFLICT OF INTEREST DECLARATION

Authors declare no conflict of interest.

## OFF‐LABEL ANTIMICROBIAL DECLARATION

Authors declare no off‐label use of antimicrobials.

## INSTITUTIONAL ANIMAL CARE AND USE COMMITTEE (IACUC) OR OTHER APPROVAL DECLARATION

Approved by Stockholms Djurförsöksetiska Nämnd, Dnr4599‐202.

## HUMAN ETHICS APPROVAL DECLARATION

Authors declare human ethics approval was not needed for this study.

## Supporting information


**Data S1.** Supporting Information

**Table S1.** Statistics of WGS data of rickets and control pugs.
