# Supplementary material for: Mutations in the CYP27B1 gene cause vitamin D dependent rickets in pugs
Source: J Vet Intern Med. 2023 Jun 9;37(4):1507–13. doi: 10.1111/jvim.16791 (PMC10365047; doi:10.1111/jvim.16791)
Supplement: Supplementary file 1 — Data S1. Supporting Information [file JVIM-37-1507-s001.pdf]

Supplementary Material

Genomic DNA isolation:

DNA was extracted from whole blood using the Macherey-Nagel, Nucelospin Blood Kit (740951). The protocol was followed except that the DNA was eluted in 100 µl of water instead of BE buffer, while 50 µl water were incubated for 3 min at room temp and then centrifuged for 1 min at 11,000 x g and a second time with 50 µl water incubated for 3 min at room temp and then centrifuged for 1 min at 11,000 x g. Concentration was measured on Nanodrop ND-100 spectrophotometer (ThermoFisher).

Genome sequencing and variant calling:

Libraries of Pug 1.1 and Pug 2 were constructed according to the protocol (Illumina DNA PCR-Free Library Prep, Tagmentation) and sequenced on Nova Seq 6000 system at SNP&SEQ Technology platform in Uppsala University (Sweden). In addition, whole genome sequencing (GWAS) data of 20 whole genome sequenced pugs were extracted from SRA database, and used as controls during the variants screening (Supplementary table 1). The paired reads were mapped to dog UU_cFam_GSD_1.0 (canfam4)[1] with BWA-men2 (v2.1)[2].

The alignments were sorted by the chromosomal coordinates and indexed by Samtools (v1.1.5)[3]. PCR and optical duplicate reads were detected and marked by the MarkDuplicate from Picard (v2.275; http://broadinstitute.github.io/picard/).

The variations were detected from each sample by HaplotypeCaller from GATK (v4.2.0.0)[4]. Genotypes of samples were called from a joint analysis by CombineGVCFs and GenotypeGVCFs of GATK. Only biallelic SNPs and indels were kept for the following analysis.

Quality control was performed of SNPs and INDELs using “hard-filtering” parameters[5] “QD < 2.0 || FS > 60.0 || MQ < 40.0 || MQRankSum < −12.5 || ReadPosRankSum < −8.0” and “QD < 2.0 || FS > 200.0 || ReadPosRankSum < −20.0”, respectively. In addition, the genotypes were required to be called in both Pug 1.1 and Pug 2, and in at least of 18 of 20 control pugs. Functional effects of the variants were predicted by SnpEFF (v4.3t)[6] with annotation from NCBI.

PCR and Sanger sequencing:

PCR was performed with AmpliTaq Gold DNA polymerase (ThermoFisher, table 1) followed by PCR clean-up using exonucelase, Fast AP-CIAp, buffer FastAP (ThermoFisher, table 2). Preparation of samples for Sanger sequencing (table 3), there after the samples were sent to Eurofins Genomics (Germany), for Sanger sequencing.

Table 1.

PCR	Volume (µl)
10x RB	2
25 mM $MgCl_2$	1.2
20 mM dNTP	0.2
Taq (AmpliTaq Gold)	0.14
Forward primer 10 µM	0.5
Reverse primer 10 µM	0.5
DNA (30-40 ng)	1
H_2O	14.46
Total	20 µl

Table 2.

ExoCiap	Volume (µl)
Exonuclease 20 U/µl (ENO582)	0.5
Fast AP-CIAP 1U/µl (EFO651)	0.5
Buffer FastAp	1
PCR product	5
Total	7 µl

Table 3.

Sanger Seq	Volume (µl)
ExoCiap PCR	3
10 µM primer (F or R)	2
H_2O	12
Total	17 µl

Sanger Sequencing results showing the homozygous chr10:2182971G>T mutation, the wild type (non-mutated), and the heterozygous mutation.

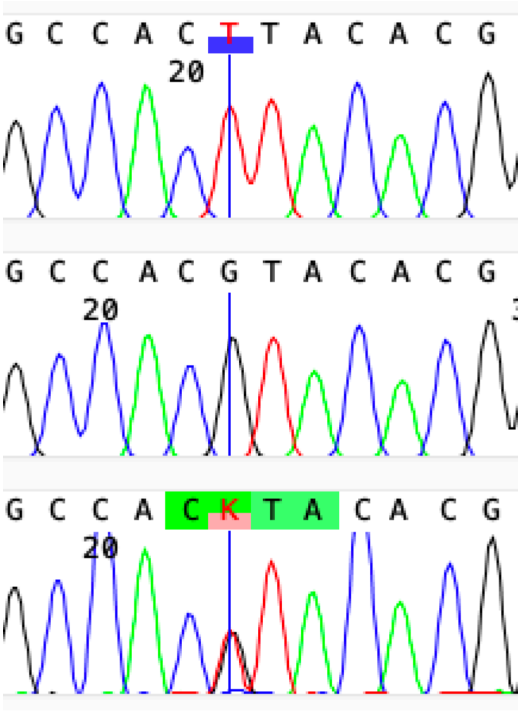

Mutant (TT)

Wild type (GG)

Heterozygot (K= GT)

References:

1. Wang C, Wallerman O, Arendt ML, et al. A novel canine reference genome resolves genomic architecture and uncovers transcript complexity. Commun Biol 2021;4:185.
2. Vasimuddin M, Misra S, Li H, et al. Efficient Architecture-Aware Acceleration of BWA-MEM for Multicore Systems. In: 2019 IEEE International Parallel and Distributed Processing Symposium (IPDPS) 2019;314-324.
3. Danecek P, Bonfield JK, Liddle J, et al. Twelve years of SAMtools and BCFtools. Gigascience 2021;10.
4. McKenna A, Hanna M, Banks E, et al. The Genome Analysis Toolkit: a MapReduce framework for analyzing next-generation DNA sequencing data. Genome Res 2010;20:1297-1303.
5. DePristo MA, Banks E, Poplin R, et al. A framework for variation discovery and genotyping using next-generation DNA sequencing data. Nat Genet 2011;43:491-498.
6. Cingolani P, Platts A, Wang le L, et al. A program for annotating and predicting the effects of single nucleotide polymorphisms, SnpEff: SNPs in the genome of Drosophila melanogaster strain w1118; iso-2; iso-3. Fly (Austin) 2012;6:80-92.
