# Supplementary material for: Mutations in the CYP27B1 gene cause vitamin D dependent rickets in pugs
Source: J Vet Intern Med. 2023 Jun 9;37(4):1507–13. doi: 10.1111/jvim.16791 (PMC10365047; doi:10.1111/jvim.16791)
Supplement: Supplementary file 2 — Table S1. Statistics of WGS data of rickets and control pugs. [file JVIM-37-1507-s002.pdf]

**Supplementary Table 1.**
Statistics of WGS data of rickets and control pugs.

The WGS data of two rickets pugs, Pug 1.1 and Pug 2, was deposited in SRA database under BioProject PRJNA911468.

| Samples | Accession | Read length | Read pairs | Percent reads aligned | Mean coverage |
|---|---|---|---|---|---|
| Pug 2 | SRR22702937 | 150 bp | 551136668 | 99.5% | 23.2 X |
| Pug 1.1 | SRR22702938 | 150 bp | 864125122 | 99.6% | 35.3 X |
| Pug_c1 | ERR2196264 | 150 bp | 813009504 | 99.5% | 44.*0* X |
| Pug_c2 | ERR2196265 | 150 bp | 824319050 | 99.8% | 43.9 X |
| Pug_c3 | ERR2196266 | 150 bp | 881463016 | 99.8% | 46.7 X |
| Pug_c4 | ERR2196267 | 150 bp | 737391208 | 99.8% | 40.1 X |
| Pug_c5 | ERR2196268 | 150 bp | 880233336 | 99.6% | 46.2 X |
| Pug_c6 | ERR2196269 | 150 bp | 758202448 | 97.9% | 41.3 X |
| Pug_c7 | ERR2196270 | 150 bp | 831189298 | 98.9% | 44.3 X |
| Pug_c8 | ERR2196271 | 150 bp | 821698002 | 98.8% | 44.0 X |
| Pug_c9 | ERR2196272 | 150 bp | 803148406 | 98.4% | 43.1 X |
| Pug_c10 | ERR2196273 | 150 bp | 775776118 | 99.6% | 42.0 X |
| Pug_c11 | ERR2196274 | 150 bp | 765725036 | 99.6% | 41.2 X |
| Pug_c12 | ERR2196275 | 150 bp | 811946908 | 99.8% | 42.9 X |
| Pug_c13 | ERR2196276 | 150 bp | 721346876 | 99.7% | 40.4 X |
| Pug_c14 | ERR2196277 | 150 bp | 711367724 | 99.7% | 39.9 X |
| Pug_c15 | ERR2196278 | 150 bp | 732579918 | 99.6% | 40.7 X |
| Pug_c16 | ERR3486153 | 150 bp | 915350230 | 97.9% | 40.1 X |
| Pug_c17 | ERR3486154 | 150 bp | 910149748 | 97.5% | 41.2 X |
| Pug_c18 | ERR3486155 | 150 bp | 873198416 | 97.4% | 39.7 X |
| Pug_c19 | ERR3486156 | 150 bp | 890195812 | 97.3% | 39.9 X |
| Pug_c20 | ERR3486157 | 150 bp | 910178494 | 97.4% | 39.7 X |
